# Comparing Long-Term Outcomes in Glomerular Disease Patients Presenting with Nephrotic Syndrome Versus Nephrotic Range Proteinuria

**DOI:** 10.3390/life14121674

**Published:** 2024-12-18

**Authors:** Gabriel Ștefan, Simona Stancu, Adrian Zugravu, Nicoleta Petre

**Affiliations:** 1 Department of Nephrology, Faculty of Medicine, University of Medicine and Pharmacy “Carol Davila”, 050474 Bucharest, Romania; simonastancu2003@yahoo.com (S.S.); adzugravu@yahoo.com (A.Z.); nicoleta.petre@gmail.com (N.P.); 2 Department of Nephrology, “Dr. Carol Davila” Teaching Hospital of Nephrology, 010731 Bucharest, Romania

**Keywords:** nephrotic syndrome, nephrotic range proteinuria, glomerular diseases, kidney biopsy, end-stage kidney disease, mortality

## Abstract

Background: Despite extensive research on proteinuria’s impact on chronic kidney disease progression, there is no direct comparison of outcomes in biopsy-diagnosed glomerular disease (GD) patients with nephrotic syndrome (NS) or nephrotic range proteinuria (NRP). Our study addresses this gap, comparing long-term outcomes between NS and NRP. Methods: We conducted a retrospective study on 240 kidney biopsy-proven GD patients, tracked from 2010 to 2015 until end-stage kidney disease (ESKD), death, or the study end in January 2022. Results: The median follow-up was 8.8 years. Diagnoses were predominantly nonproliferative (53%), proliferative (25%) nephropathies, diabetic nephropathy (12%), and paraprotein diseases (10%). NS was observed in 141 (59%) patients, presenting more frequently with arterial hypertension, higher eGFR, increased proteinuria, and dyslipidemia than NRP patients. NRP patients often had proliferative GD and diabetic nephropathy; their renal chronicity score was higher. The ESKD endpoint occurred in 35% NS and 39% NRP patients (*p* 0.4). The cohort’s mean kidney survival time was 8.2 years. In a multivariate analysis, NS, lower eGFR, a higher renal chronicity score, and diabetic nephropathy were associated with ESKD. A total of 64 patients (27%) died, 73% post-kidney replacement therapy initiation, and mostly from cardiovascular disease (63%). Mortality between proteinuria forms showed no difference. The multivariate analysis found lower eGFR, a higher Charlson comorbidity score, and diabetic nephropathy associated with mortality. Conclusions: Our study found no difference in all-cause mortality between NS and NRP in glomerular diseases. However, an adjusted analysis revealed poorer kidney survival for NS patients, emphasizing the need for personalized management to improve renal prognoses.

## 1. Introduction

Proteinuria is a key feature of glomerular diseases, often serving as both a clinical hallmark for initial diagnoses and a therapeutic target for slowing disease progression [1,2,3,4].

Heavy proteinuria of glomerular origin primarily manifests in two main forms: nephrotic syndrome (NS) and nephrotic range proteinuria (NRP). NS is defined by proteinuria higher than 3.5 g/day, resulting in hypoalbuminemia and edema, and often includes hyperlipidemia, and increased risks of a thromboembolism and infection [2,3]. On the other hand, NRP shares NS’s protein excretion level but lacks its other features and arises from heightened permeability of the glomerular basement membrane due to primary or secondary kidney diseases [1,4].

There is mounting evidence indicating that both types of proteinuria are not merely passive markers of glomerular damage but also active contributors to the progression of chronic kidney disease (CKD) [5,6,7]. Moreover, proteinuria has been linked to an increased risk of cardiovascular mortality, further expanding its significance beyond a nephrological context [8,9,10].

Although the relationship between proteinuria and CKD progression has been extensively investigated, no study has directly compared outcomes between patients diagnosed with glomerular diseases by kidney biopsy who present with either NS or NRP. These disease states’ implications for end-stage kidney disease (ESKD) and all-cause mortality outcomes warrant investigation for a more nuanced understanding of proteinuria’s impact on patient prognoses.

Therefore, given the potential prognostic significance of proteinuria in the form of NS and NRP, our study aimed to bridge the knowledge gap by providing a head-to-head comparison of long-term outcomes.

## 2. Methods

### 2.1. Study Design and Population

This is a retrospective, longitudinal study conducted on adult patients with a pathological diagnosis of glomerular disease, who underwent native kidney biopsy at Dr. Carol Davila Teaching Hospital of Nephrology between 1 January 2010 and 31 December 2015 [11].

**Figure 1:**
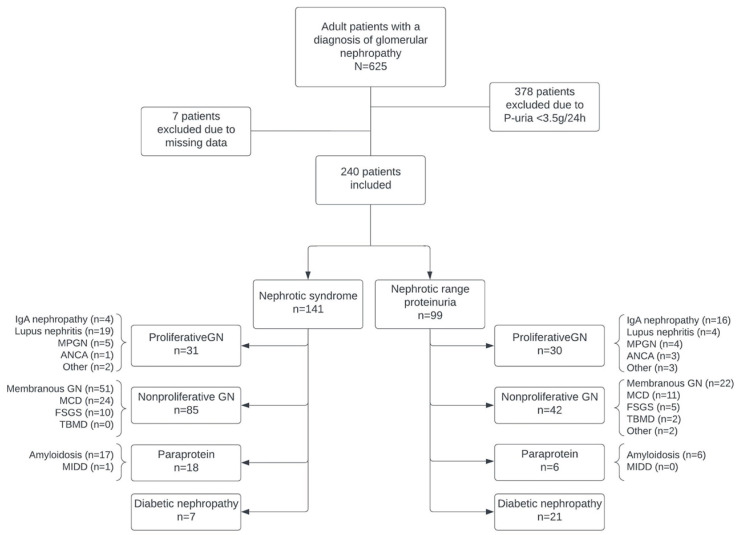
Primary clinicopathological diagnoses of the patients included in the study. n, actual number of patients in each category; ANCA, anti-neutrophil cytoplasmic antibody; FSGS, focal and segmental glomerulosclerosis; GN, glomerulonephritis; MCD, minimal change disease; MIDD, monoclonal immunoglobulin deposition disease; P-uria, proteinuria; TBMD, thin glomerular basement membrane disease.

Out of 625 patients initially identified with glomerular nephropathies, we implemented selection criteria that resulted in the exclusion of 378 patients who exhibited proteinuria levels of less than 3.5 g per day. An additional 7 patients were excluded due to incomplete data sets. Consequently, the final study cohort comprised 240 patients (Figure 1).

The study was conducted with the provisions of the Declaration of Helsinki and was approved by the local ethical committee (“Dr. Carol Davila” Teaching Hospital of Nephrology, Bucharest, Romania, approval number 2022-010). Our cohort study was conducted in strict adherence to the STROBE (Strengthening the Reporting of Observational Studies in Epidemiology) criteria (Supplementary File S1).

### 2.2. Data Collection

We collected data at the moment of kidney biopsy including age, comorbidities (measured via the Charlson comorbidity score), arterial hypertension status (defined as either blood pressure exceeding 140/90 mmHg or usage of antihypertensive drugs), inflammation markers (serum hemoglobin, C-reactive protein), lipid profile (serum cholesterol and triglycerides), serum albumin, eGFR (calculated using the four-variable CKD-EPI formula), proteinuria (measured in 24 h), hematuria, and the type of therapy used (angiotensin-converting enzyme inhibitor/angiotensin-receptor blocker—ACEI/ARB—or immunosuppression).

Of note, according to our local protocol, the adequacy of the 24 h urine collection was assessed by comparing the total creatinine in the sample to the predicted creatinine [22 − (age/9) mg/kg/day in women and 28 − (age/6) mg/kg/day in men]. Collections were considered accurate if measured/expected ratios were between 0.8 and 1.2 [1].

### 2.3. Definitions

Nephrotic syndrome was defined as a persistent excretion of urinary proteins, primarily albumin, at levels equal to or greater than 3.5 g/24 h, and a serum albumin concentration below the standard limit, less than 3.5 g/dL [1]. We used the <3.5 g/dL cut-off for hypoalbuminemia to capture a broader spectrum of patients with clinically relevant nephrotic syndrome, including those at earlier stages who may still experience significant complications. This higher threshold ensures comparability with prior studies and aligns with the variability seen in real-world clinical practice, where hypoalbuminemia often manifests across a continuum [1].

Nephrotic range proteinuria was synonymous with the proteinuria observed in nephrotic syndrome, but with serum albumin higher than 3.5 g/dL [1].

It is important to note that the proteinuria defined in these terms did not result from lymphatic obstruction, id est, chyluria, or from a defective reabsorption of normally filtered proteins by the proximal tubular cells (Fanconi syndrome, proximal tubular dysfunction) [1].

### 2.4. Pathological Measurements

For each kidney biopsy specimen, light microscopy, immunofluorescence, and electron microscopy were routinely performed. We utilized the chronicity scoring system for kidney biopsy proposed by Sethi et al. and validated in various populations of glomerular diseases [12,13]. This scoring system provides a semi-quantitative scale for evaluating glomerulosclerosis (0–3), interstitial fibrosis (0–3), tubular atrophy (0–3), and arteriolosclerosis (0–1). The overall chronicity score was computed by adding the individual scores for each kidney compartment [12]. Kidney biopsies were reviewed by a team of experienced renal pathologists within our department. To ensure consistency, a subset underwent a secondary review by a senior pathologist, and regular consensus meetings were held to address and resolve any discrepancies.

### 2.5. Study Endpoints

The primary endpoint of this study was the development of end-stage kidney disease (ESKD), defined as the initiation of dialysis or receipt of a kidney transplant. Patients were considered to have reached this endpoint when either event was recorded in the Romanian Renal Registry.

The secondary endpoint was all-cause mortality, assessed by documenting the total number of deaths, irrespective of cause, within the cohort during the follow-up period. All patients were followed from the start of the study (id est, moment of kidney biopsy) until 1 January 2022.

### 2.6. Statistical Analysis

Continuous variables were presented as either the mean or median accompanied by the interquartile range (IQR), following an assessment of normality using the Shapiro–Wilk test. Categorical variables were summarized as percentages.

Group differences were evaluated using Student’s *t*-test or the Mann–Whitney test, depending on the distribution characteristics of the variables. For categorical data, the Chi-square test was applied to determine statistical significance.

Event-free survival probabilities were analyzed using the Kaplan–Meier method, with group comparisons conducted via the log-rank test. Independent predictors for progression to ESKD and lack of remission were identified through univariate and multivariate analyses using Cox proportional hazard models, with results reported as hazard ratios (HRs) and 95% confidence intervals (CIs). All tests were two-tailed, and a *p*-value less than 0.05 was deemed significant, indicating strong evidence against the null hypothesis. Statistical analyses were conducted using SPSS version 26 software (SPSS, Chicago, IL, USA).

## 3. Results

### 3.1. Patients’ Characteristics

The study population included 240 patients (65% male) with kidney biopsy-proven glomerular diseases who were followed for a median of 8.8 (95%CI: 8.5, 9.0) years. At the time of diagnosis, their median age was 50 years, the median eGFR was 58 mL/min/1.73 m^2^, the median proteinuria was 5.3 g/day, 50% had arterial hypertension, and 12% had type 2 diabetes mellitus (Table 1).

Of the study participants, 59% (141 patients) were diagnosed with nephrotic syndrome, while 41% (99 patients) had nephrotic range proteinuria.

The main primary clinicopathological diagnosis was nonproliferative glomerulopathies (53%), followed by proliferative glomerulopathies (25%), diabetic nephropathy (12%), and paraprotein diseases (10%) (Figure 1). The most frequent disease in the nonproliferative glomerulopathies group was membranous nephropathy (n = 73), followed by minimal change disease (n = 35) and focal and segmental glomerulosclerosis (n = 15). Meanwhile, in the proliferative glomerulopathies group, lupus nephritis (n = 23) prevailed, followed by IgA nephropathy (n = 20) and membranoproliferative glomerulonephritis (n = 9) (Figure 1).

On histopathological assessment, the median total renal chronicity score was 1 and glomerulosclerosis was present in 41% cases, interstitial fibrosis in 31%, tubular atrophy in 23%, and arteriosclerosis in 21% (Table 1).

Overall, during the follow-up, renin–angiotensin system inhibitors and immunosuppression were used in 55% and 75%, respectively (Table 1).

### 3.2. Nephrotic Syndrome Versus Nephrotic Range Proteinuria

Patients who presented with NS more frequently had arterial hypertension, a higher eGFR, increased proteinuria, and more pronounced dyslipidemia (Table 1).

The distribution of primary clinicopathologic diagnoses differed significantly between the two groups. Although nonproliferative glomerulopathies were the most common etiology in both groups, patients with NRP more often had proliferative glomerulopathies and diabetic nephropathy (Table 1).

Chronic lesion scores, including those for glomerulosclerosis, interstitial fibrosis, tubular atrophy, arteriosclerosis, and the overall renal chronicity score, were significantly higher in the NRP group compared to the NS group (Table 1).

The use of angiotensin-converting enzyme inhibitors (ACEIs/ARB) was similar between the two groups. However, immunosuppression therapy was more often administered to patients with NS (Table 1).

### 3.3. Kidney Survival

The ESKD endpoint occurred in 35% of the NS group and 39% of the NRP group (*p* 0.4).

The mean kidney survival time of the cohort was 8.2 (95%CI: 7.6, 8.8) years; survival at 1, 3, 5 and 10 years was 88%, 75%, 70%, and 60%, respectively.

**Figure 2:**
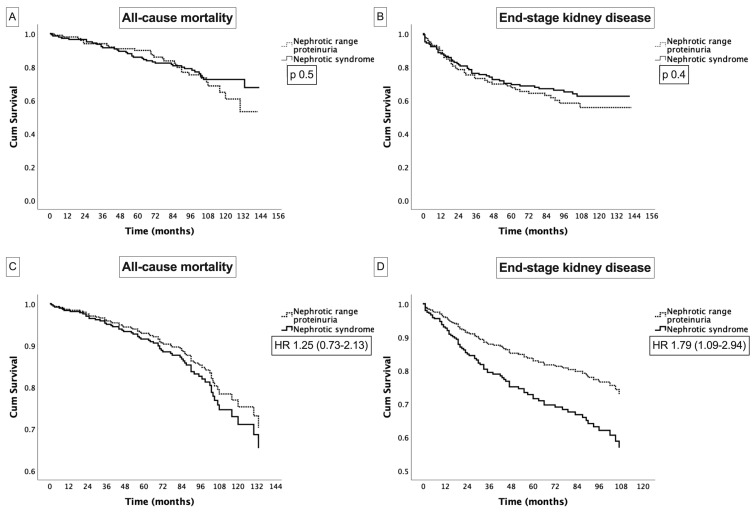
(**A**). Nonadjusted Kaplan–Meier survival curves for nephrotic range proteinuria versus nephrotic syndrome for all-cause mortality; (**B**). Nonadjusted Kaplan–Meier survival curves for nephrotic range proteinuria versus nephrotic syndrome for kidney survival (ESKD); (**C**). Adjusted survival curve for all-cause mortality (Table 2); (**D**). Adjusted survival curve for kidney survival (Table 2).

In the Kaplan–Meier analysis, patients with NS showed a similar mean kidney survival time to those with NRP (8.4 (95%CI: 7.6, 9.1) versus 8.0 (95%CI: 7.0, 8.9) years, *p* 0.4) (Figure 2B). However, in the multivariate Cox regression analysis, factors including NS, a lower eGFR, a higher total renal chronicity score, and diabetic nephropathy as a glomerular disease were all significantly and independently associated with ESKD (Table 2, Figure 2D).

### 3.4. Patient Survival

In total, 64 patients (27%) died during the follow-up period, with the majority of these deaths—47 (73% of total deaths)—occurring after the initiation of kidney replacement therapy. The leading cause of death was cardiovascular disease (63%), followed by infectious diseases (22%), oncological diseases (11%), gastroenterological diseases (3%), and neurological diseases (1%). However, mortality rates were similar between both groups (26% in the NS group and 28% in the NRP group, *p* 0.6).

The mean survival time of the cohort was 9.9 (95%CI: 9.4, 10.3) years, with survival rates at 1, 3, 5, and 10 years being 98%, 93%, 88%, and 68%, respectively.

The Kaplan–Meier analysis revealed no significant difference in survival time between the two groups: 9.9 (95%CI: 9.4, 10.5) years for NS versus 9.7 (95%CI: 9.0, 10.3) years for NRP (*p* 0.5) (Figure 2A). In the multivariate Cox regression analysis, a lower eGFR, a higher Charlson comorbidity score, and diabetic nephropathy were independently associated with mortality (Table 2, Figure 2C).

## 4. Discussion

In the present study, we aimed to compare the long-term outcomes of patients diagnosed with glomerular diseases presenting as nephrotic syndrome or nephrotic range proteinuria.

The main results of our study are that (i) there was no difference in terms of all-cause mortality between the two forms of proteinuria present at diagnoses; (ii) in our analysis of kidney survival rates, a differential trend emerged between patients with nephrotic syndrome and those with nephrotic range proteinuria. Though the initial Kaplan–Meier method analysis revealed no significant difference, the adjusted analysis considering risk factors showed poorer kidney survival among patients with nephrotic syndrome.

One possible explanation for these findings could be that nephrotic syndrome is not just characterized by high levels of proteinuria but also by a host of other features such as hypoalbuminemia, hyperlipidemia, progressive kidney failure, increased risk of infection, and thrombosis [4,14,15,16]. These additional manifestations indicate a more advanced disease state and possibly more extensive kidney damage, thereby leading to a higher risk of poor renal outcomes. Moreover, in patients with hypoalbuminemia due to NS, kidney dysfunction typically presents in two ways: failure to regulate sodium and fluid levels, and a decrease in the ultrafiltration ability of glomerular capillary walls, which subsequently leads to a drop in the GFR [17].

In our study, the risk factors that were controlled in the multivariate analysis, such as age, comorbidities, or baseline kidney function, could exert a more pronounced effect on the progression of renal disease in patients with NS. The interaction of these factors could result in a more rapid deterioration of a vulnerable kidney function, thus explaining the poorer renal survival observed in these patients.

Kolb et al. analyzed 522 Scottish Renal Biopsy Registry patients, diagnosed with nephrotic syndrome between 2014 and 2017, and linked their records to national mortality data [10]. They reported a 21% mortality rate over a median follow-up period of 2.4 years, a figure notably higher than the 26% mortality rate we observed in our own study during a median follow-up of 8.8 years [10]. Age and secondary causes of NS were primary mortality predictors in their cohort [10], whereas our study associated mortality with a higher comorbidity index, lower baseline eGFR, and specific histological diagnoses, particularly diabetic nephropathy. Moreover, 15% of patients from Kolb et al.’s study progressed to end-stage kidney disease during follow-up [10], contrasted with 28% in our long-term follow-up single-center study. The difference in outcomes may result from the differing methodologies of the studies; the Scottish study was a nationwide examination, whereas our research was conducted within a single center. Nevertheless, both studies’ results indicate the importance of prioritizing patient care and ensuring timely preparation for renal replacement therapy and advanced care planning for patients with NS.

Our results also align with Li et al.’s study on Chinese IgA nephropathy patients, which utilized propensity score matching to compare the long-term outcomes of 57 patients with NRP and 59 patients with NS [18]. After a median follow-up period of 18.5 months, similar percentages of patients in both groups reached the primary outcome (22% NS vs. 17.5% NRP), which included a doubling of baseline serum creatinine, a 50% reduction in eGFR, the onset of ESKD (eGFR < 15 mL/min/1.73 m^2^), or death [18].

Patients with NRP often have secondary forms of focal segmental glomerulosclerosis (FSGS), such as reflux nephropathy, rather than primary nephrotic disorders like membranous nephropathy, primary FSGS, or minimal change disease [8]. This observation is consistent with our study, where patients with NRP frequently showed conditions such as diabetic nephropathy, IgA nephropathy, or FSGS.

The Ramipril Efficacy in Nephrology (REIN) study was the first to comprehensively examine the impact of proteinuria on the advancement of kidney disease. This trial revealed that among 352 patients suffering from proteinuric nephropathies of various origins, those with elevated levels of proteinuria at the onset of the study were correlated with a faster decline in eGFR and a more rapid progression to ESKD [5,19]. A pooled analysis involving 2387 CKD patients across 11 trials further confirmed proteinuria’s predictive pathogenic role. It found that regardless of treatment, short-term proteinuria changes aligned with long-term outcomes, whereas no change in proteinuria indicated no long-term benefit [20].

In adults, type 2 diabetes frequently results in NRP, leading to rapid kidney function loss, early ESKD, increased healthcare usage, and cardiovascular disease [21,22]. While therapeutic options are limited, angiotensin-converting enzyme inhibitors or angiotensin-receptor blockers have been shown to be effective [7,23]. Additionally, the EMPA-REG OUTCOME trial suggests that empagliflozin, an SGLT2 inhibitor, may slow kidney disease progression in these high-risk patients [6].

Given our study’s finding of poorer renal survival in patients with NS, it is important to implement antiproteinuric strategies proven to be effective in managing NRP for these patients as well.

In our cohort, patients with NRP exhibited lower eGFR and higher chronic lesion scores on kidney biopsy than those with NS, suggesting a distinct mechanism driving NRP.

Growing evidence indicates that most kidney diseases progress to renal failure due to functional adaptations in the kidney following an initial nephron loss from the primary disease process. These adaptations initially counteract nephron loss but eventually become harmful. This malfunction results in protein ultrafiltration, triggering inflammatory and apoptotic pathways, which further exacerbate kidney damage [24]. Excessive proteinuria can also injure podocytes and promote the myofibroblast differentiation of mesangial cells, and lead to toxicity to the proximal tubules [24,25,26,27]. Both in vitro and in vivo protein overload increases the production of vasoactive and inflammatory mediators, leading to abnormal extracellular matrix accumulation in the interstitium and causing interstitial fibrosis [28]. The resultant inflammation stimulates macrophage and lymphocyte recruitment, potentially transforming interstitial cells into myofibroblasts [29,30]. In patients with NRP, the lack of hypoalbuminemia could be due to the absence of certain cytokines, such as tumor necrosis factor and interleukin-1, which are typically released in those with primary nephrotic syndrome [31,32]. Notably, these cytokines have been identified to directly inhibit hepatic albumin synthesis [31].

Interestingly, ESKD occurred five times more frequently than pre-ESKD deaths in our study population (37% versus 7%). Additionally, mortality nearly tripled following the initiation of kidney replacement therapy. This suggests that mortality in patients with glomerular diseases presenting as NS and NRP arises post-ESKD onset, contrasting with larger studies on “general” CKD where mortality risk rises even before reaching ESKD [33,34,35]. The reason for this discrepancy between NS/NRP patients and other CKD patients remains unclear. It might be attributable to factors such as younger age and fewer extrarenal organ involvements or less severe comorbidities at diagnoses, allowing these patients to withstand the progression of CKD and only succumbing during ESKD. A closer medical follow-up in autoimmune diseases could also be a contributing factor. The higher rate of progression to ESKD compared to mortality observed in our cohort aligns with findings from other glomerular disease studies, such as the IgA nephropathy cohort, highlighting the unique disease-specific risks and the impact of specialized care and follow-up provided in dedicated centers like ours [36]. However, these findings challenge the universal applicability of mortality risk observed in generalized CKD studies, which often group diverse kidney diseases under the generic CKD label.

Our research provides significant insights into NS and NRP with a median follow-up of 8.8 years, marking the longest observation period in any comparative study on this topic to date. The observed mean kidney survival time of 8.2 years can be indicative of the point in the disease trajectory at which biopsies tend to occur within our institution. It is worth highlighting that this study was conducted in a unicentric setting. The timing of biopsy presentations might be influenced by specific patient demographics or clinical protocols inherent to our center. As we build on this foundational work, there is an exciting potential for multi-center studies to further explore and validate these findings, offering a broader and more diverse understanding of NS and NRP disease progression.

Our study has several limitations that should be acknowledged when interpreting the results. First, as a single-center observational study, the findings are inherently limited in scope and generalizability. The patient population and treatment protocols at our center may differ from those of other institutions, making it challenging to apply our results universally to all patients with NRP or NS.

Second, the comparison between NRP and NS was conducted solely at the time of presentation, specifically at the time of kidney biopsy. Consequently, our analysis does not account for potential changes in disease status or treatment responses over time, which could affect long-term outcomes. Moreover, our study’s sample size restricted our ability to conduct in-depth subgroup analyses for each primary glomerular disease, emphasizing the need for larger future studies to comprehensively address these subgroup variations.

Additionally, given the observational nature of our research at the point of kidney biopsy (diagnosis), there exists the possibility that biopsies for the NS and NRP groups were performed at different stages of kidney diseases. The groups exhibited notable disparities in aspects such as the arterial hypertension presence, utilization of immunosuppressives, eGFR, proteinuria amounts, and renal chronicity scores, which indeed may compromise their direct comparability. Another limitation of our study is the potential for diagnostic overlap between MCD and FSGS, as a third of MCD cases presented with nephrotic range proteinuria, which is uncommon for MCD and may raise concerns about misclassification. Furthermore, given the pioneering nature of our exploration on the topic, our study essentially serves as a hypothesis-generating endeavor. The absence of an external validation cohort in this study underscores the importance and necessity of follow-up research to validate our findings across diverse patient cohorts.

Despite these limitations, we believe that our study provides valuable insights and a basis for further research into the outcomes and management of patients with NRP and NS.

In comparing long-term outcomes of nephrotic syndrome and nephrotic range proteinuria in glomerular diseases, our study found no difference in all-cause mortality. However, the adjusted analysis revealed poorer kidney survival for nephrotic syndrome patients, underscoring the need for personalized management strategies to improve their renal prognosis.

## Figures and Tables

**Table 1:** Patients’ characteristics according to the type of proteinuria (nephrotic syndrome versus nephrotic range proteinuria).

	Total N = 240	Nephrotic Syndrome n = 141	Nephrotic Range Proteinuria n = 99	*p*
Age, yr	50 (39–62)	50 (37–63)	48 (40–61)	0.9
Men, %	65	61	70	0.1
Primary clinicopathologic diagnosis, %				<0.001
Proliferative glomerulopathies	25	22	30	
Nonproliferative glomerulopathies	53	60	42	
Paraprotein	10	13	6	
Diabetic nephropathy	12	5	22	
Charlson comorbidity score	2 (2–3)	2 (2–3)	2 (2–3)	0.8
Arterial hypertension, %	50	61	43	<0.001
eGFR, mL/min	58.0 (34.4–77.1)	64.9 (39.1–83.6)	51.5 (29.0–66.4)	<0.001
Serum creatinine, mg/dL	1.3 (1.0–2.0)	1.2 (0.9–1.8)	1.4 (1.1–2.5)	<0.001
Proteinuria, g/24 h	6.0 (4.5–8.1)	6.4 (4.8–9.0)	5.4 (4.0–7.1)	<0.01
Hematuria, mm^3^	35 (5–190)	35 (5–190)	30 (5–180)	0.6
Hemoglobin, g/dL	13.0 (11.3–14.6)	13.1 (11.2–14.7)	12.3 (11.3–14.5)	0.4
Serum albumin, g/dL	3.3 (2.7–3.8)	2.9 (2.5–3.1)	3.9 (3.7–4.1)	<0.001
C-reactive protein, mg/L	3.0 (1.0–7.0)	3.0 (2.0–7.0)	3.0 (1.0–6.0)	0.2
Serum cholesterol, mg/dL	273 (228–336)	316 (248–400)	251 (200–289)	<0.001
Serum triglycerides, mg/dL	211 (141–303)	226 (152–320)	184 (123–270)	<0.001
Chronic lesion score				
Glomerulosclerosis, % (0/1/2/3)	59/16/17/8	69/17/10/4	44/14/27/15	<0.001
Interstitial fibrosis, % (0/1/2/3)	69/20/9/2	77/16/6/1	57/25/14/4	<0.01
Tubular atrophy, % (0/1/2/3)	77/14/6/3	86/10/3/1	64/20/11/5	0.001
Arteriosclerosis, %	21	16	29	0.01
Total renal chronicity score	1 (0–3)	0 (0–1)	2 (0–5)	<0.001
Medication, %				
ACEI/ARB	55	51	62	0.1
Immunosuppression	75	82	65	<0.01
ESKD endpoint, %	37	35	39	0.4
Death, %	27	26	28	0.6

ACEI/ARB, angiotensin-converting enzyme inhibitor/angiotensin-receptor blocker; ESKD, end-stage kidney disease; eGFR, estimated glomerular filtration rate.

**Table 2:** Multivariate Cox regression analysis for determining risk factors at diagnosis for end-stage kidney disease and all-cause mortality.

	End-Stage Kidney Disease		All-Cause Mortality	
	HR (95%CI)	*p*	HR (95%CI)	*p*
Type of proteinuria				
Nephrotic range proteinuria	Reference		Reference	
Nephrotic syndrome	1.79 (1.09–2.94)	0.02	1.25 (0.73–2.13)	0.4
eGFR (mL/min)	0.97 (0.96–0.98)	<0.001	0.98 (0.97–0.99)	<0.001
Comorbidity score	1.11 (0.93–1.32)	0.2	1.38 (1.17–1.64)	<0.001
Total renal chronicity score	1.12 (1.02–1.23)	0.01	1.02 (0.90–1.16)	0.6
Glomerular disease		<0.001		0.01
Diabetic nephropathy	Reference		Reference	
Proliferative GN	0.39 (0.18–0.88)	0.02	0.48 (0.20–1.14)	0.09
Nonproliferative GN	0.25 (0.10–0.62)	0.03	0.50 (0.19–1.30)	0.1
Paraprotein	1.72 (0.72–4.09)	0.2	1.38 (0.51–3.74)	0.5
ACEI/ARB				
No	Reference		Reference	
Yes	0.74 (0.47–1.15)	0.1	1.28 (0.76–2.18)	0.3
Immunosuppression				
No	Reference		Reference	
Yes	0.68 (0.37–1.24)	0.2	0.60 (0.31–1.14)	0.1

ACEI/ARB, angiotensin-converting enzyme inhibitor/angiotensin-receptor blocker; CI, confidence interval; eGFR, estimated glomerular filtration rate; GN, glomerular nephropathy; HR, hazard ratio.

## Data Availability

The data underlying this article will be shared on reasonable request to the corresponding author.

## Supplementary Materials

The following supporting information can be downloaded at: https://www.mdpi.com/article/10.3390/life14121674/s1, File S1: STROBE Statement—Checklist of items that should be included in reports of cohort studies.
